# Monthly Summary

**Published:** 1888-05

**Authors:** 


### Monthly Summary.

---

FORMER ILLIBERALITY IN THE PROFESSION.—Dr. Dwinelle, of New York, one of the oldest practitioners of the profession, says: When I first essayed to enter the profession I was snubbed at every point. I could not get information from any one unless I paid a price altogether beyond my means. I stopped at a city in the northern part of this State, not a thousand milcs from Albany, and went in search of information. I had a limited amount of means in my pocket, and was willing to expend it for that purpose, though I ought not to have been willing to expend a cent. I was kept in the reception room, and when I made a suggestion that I would like to go into the operating room or laboratory and see what was going on there I was met with the statement that it would require from five hundred to a thousand dollars to gain the desired admittance. I was treated in the most illiberal manner, and, poor as I was, I left the house with a degree of righteous indignation that I think was worthy of a saint; and when I got outside I looked up, and though I did not pronounce a curse on that house, I did promise myself that I would see the day when those two dentists would sit at my feet and learn wisdom. Years afterward, when I had attained some reputation and was located in this city, I was gratified to receive a letter from these gentlemen soliciting the privilege of coming to my office and seeing my method of operating with crystal gold and cohesive foil, which was new to them. I immediately responded, cordially inviting them to come. They did so, and I entertained them for the most of two days, to their great satisfaction, and when they were leaving they proposed to pay me for my time and instruction. I repelled this proposition almost with indignation, as being contrary to the true spirit of professional courtesy, and reminded them that it would be both illiberal and degrading to make a traffic of what should be free to us all. They departed, seemingly under a deep sense of obligation to me, and I could not resist the conviction that they were impressed with the difference in the treatment they had received and that which they had extended to me years before.—*Cosmos.*

---

WASTE.—The complete erasure of the word "waste" from the dictionaries, at all events in so far as it has any relation to industrial products, if not quite an accomplished fact, is undoubtedly becoming more and more imminent; and we may thank the chemists of this generation for teaching us how to recover and utilize innumerable substances which, our grandfathers in their ignorance, threw away Thirty years ago the manufacturers of iron, gas, and chemicals everywhere neglected all but the prime objects of their industries, whereas to-day, on the system of taking care of the pennies and allowing the pounds to take care of themselves, competition has induced us to regard our many by-products as so many integral parts or branches of each enterprise. If the intelligent men who have "gone before," and who were looked on by their contemporaries as wise in the generation, could by any chance reappear among us, we might conduct them to our gas works, and with a certain pride explain the origin of our sulphate of ammonia, our aniline dyes, and our hundred other extracts from coal tar. From the contemplation of gas we would turn with them to some of our smelters and furnaces, and point to the mineral wood, the cement, the glassware, the pottery, the fire bricks, and the fertilizer, all derived from our furnace slag, and finally, entering a great chemical works, we would show them how the once devastating gases, so fatal to life and vegetation, are no longer sent free into the air, but are condensed and transformed into staple articles of trade, and how by an ingenious and, to them, undreamed of process; we extract the precious metals from our exhausted sulphur ores. To their wondering question, "How can these things be?" we might reply that all these marvels result from a modern and enlightened policy, which, in many countries, has fostered every species of research in every branch of science, encouraged great minds to ponder over and gradually unravel the mysteries of nature and stimulated a general thirsting for that knowledge which, properly applied, must ever ameliorate our condition in this 'vale of tears."—*The Age of Steel.*

---

MAKE YOUR WORK A PLEASURE.—We feel as awkward in speaking on this subject as when lecturing on intemperance; we have never experienced the misery of intemperance and therefore can only speak as an observer. So we have no experience in having an unpleasant vocation, and therefore can speak only as an observer. We have been so fortunate as always to have had work that pleased us.

But we believe, if we were ever so unfortunate as to have a work that was irksome, we would forthwith look for something pleasurable in it to relieve its irksomeness. And they say "what we look for we usually find." It is something like the weather. There are persons who are never satisfied with it. If a day does chance to suit them, it is looked on as "a weather breeder"—a sure precurser of a storm. We said to a friend the other day who was complaining of the rain, that we had not seen a day of bad weather for twenty-five years. "O, well, said he, "I suppose it is wrong to be always fretting about the weather, we have got to take it as it comes." But even this is not the spirit in which we should view either our work or the weather. We should not only be willing to tolerate it, but to meet it with cheerfulness, and to enjoy it. The weather is just right. It must be, for it represents God's wisdom and goodness; and the better we live in harmony with His laws, the clearer we shall see its adaptation to the world's best good. Besides, if we do not like the weather of one section, He has given us the means of locomotion to take us to another section, and surely we ought to be pleased somewhere. So with our work; it is given to us for our greatest good, and we have our choice of such a great variety, it is a pity if one of its many phases cannot suit us; generally, if it does not, the fault is within ourselves.—*Items of Interest.*

---

CLEANING THE TEETH.—Dentists are daily committing the error of not instructing their patients in regard to the proper methods of cleansing their mouths—brushing, picking, rinsing with warm water after meals and at night before going to bed. Our observations must show that people who do those things faithfully, have little or no dentistry to do. It is astonishing what ignorance exists among people of all classes and conditions, as to what cleanliness of the mouth means. They will tell you frankly that they do not brush their mouths as well as they ought to, for they did not know they were going to be examined and when you looked, *you really thought so*, and the second thought was *probably not for a month.* Cleanly habits are part of an individual's education and can be formed only in childhood. Too much care cannot be bestowed on the subject for the little ones. Each individual must see it thoroughly done—have it done for him and experience having it well rubbed in with a brush. Not much dentifrice of any kind is needed—small quill tooth picks are best, narrow strips of rubber dam for spaces the quill will not clean. Water used frequently for rinsing, with a motion of the tongue on all the surfaces of the teeth and gums, lingual, palatal, labial and buccal. So much for preventive dentistry which should be our highest aim.— *W. N. Morrison.*

SWEETS constitute an important part of our diet. Children are bribed to do plain duties, by promises of more sweetmeats. Drinks which would have been sickeningly sweet to a palate of years ago, are daily demanded by children of our day. Syrups to excess are used daily in all families. Milk and eggs are ruined in their healthful simplicity by the addition of sugar and lemons; and other mixtures are made that deprive the teeth of use in mastication and furnish the secretions of an abnormal quality favoring destruction by decay.

I have watched with much interest and sadness the teeth of children in families where much sugar was used, and invariably their teeth were bad. One family, relatives of mine, used four barrels of sugar a year. When adult life was reached, there was not one mouth with the full quota of teeth —all had extensive fillings made, and two of the young ladies have their mouths full of crockery substitutes. Employees of long service in confectionery shops and candy factories are also affected in the same manner. It is the excessive use of these articles, simple in themselves, which is doing the irreparable hereditary damage.— *W. N. Morrison.*

---

THE PHILOSOPHY OF A COLD.—Dr. Frank Woodbury, a good authority on colds, Professor of Materia Medica at the Medico-Chirurgical College, of Philadelphia, recently lectured at the social meeting of the Alumni Association of the Philadelphia College of Pharmacy. His subject was "The Philosophy of a Common Cold," and the practical result of his remarks may be summed up thus: The way to cure a cold is to cure the patient, and the time to cure the patient is before he gets the cold. In other words, the proper way is to abolish the "catarrhal state," and the catarrhal state of the system is that in which it is favorable for the catching of a cold. There is a susceptibility by some to take cold, while there is resistance by others. Of a large audience leaving a heated hall and entering cold air outside, some take cold while others do not. The reason why all do not take cold is because they are not susceptible. Susceptibility to cold, he said, is probably due to impairment of nutrition. Physicians find that after a period of exhaustive work they are liable to an attack of cold; but if they be in good health they pass through the same conditions with impunity. A celebrated physician, he said, attributed this catarrhal state to indolence and luxury. "In the sweat of thy face shalt thou eat bread." There is no royal road, said the lecturer, to the plow-boy's appetite, and to have the plow-boy's appetite you must do the plow-boy's work.

The state of the skin is an important part of the catarrhal state. Some people sleep in the same underclothing that they have worn during working hours. The tendency of this is to convert the skin into something like a mucous membrane; it ceases to be a protective covering. With such people the clothes are a part of the body. Benjamin Franklin advocated the air bath. The speaker said that when people came to him suffering with a cold in the upper air passages and bronchial tubes his care is to harden the skin, and for this purpose he recommends the use of the flesh brush, exposure to the air, the cold douche, frequent change of underclothing and physical exercise.

---

IN FAVOR OF A VEGETABLE DIET.—All the heavy work of the world is not done by men who eat meat. The Roman soldiers, who built such wonderful roads, and carried a weight of armor and luggage that would crush the average farm hand, lived on coarse brown bread and sour wine. They were temperate in diet and regular in exercise. The Spanish peasant works every day and dances half the night, yet eats only his black bread, onion rnd watermelon. The Smyrna porter eats only a little fruit and some olives, yet he walks off with his load of a hundred pounds. The coolie, fed on rice, is more active, and can endure more than the negro fed on fat meat.

---

MERCURY is one of the best elements in which to plunge the points of dental instruments to harden them. They should of course, be first brought to a cherry-red.

